# Investigating speed-safety association: Considering the unobserved heterogeneity and human factors mediation effects

**DOI:** 10.1371/journal.pone.0281951

**Published:** 2023-02-21

**Authors:** Habibollah Nassiri, Seyed Iman Mohammadpour

**Affiliations:** Civil Engineering Department, Sharif University of Technology, Tehran, Iran; Al Mansour University College-Baghdad-Iraq, IRAQ

## Abstract

The relationship between mean speed and crash likelihood is unclear in the literature. The contradictory findings can be attributed to the masking effects of the confounding variables in this association. Moreover, the unobserved heterogeneity has almost been criticized as a reason behind the current inconclusive results. This research provides an effort to develop a model that analyzes the mean speed-crash frequency relationship by crash severity and type. Also, the confounding and mediation effects of the environment, driver, and traffic-related attributes have been considered. To this end, the loop detector and crash data were aggregated daily for rural multilane highways of Tehran province, Iran, covering two years, 2020–2021. The partial least squares path modeling (PLS-PM) was employed for crash causal analysis along with the finite mixture partial least squares (FIMIX-PLS) segmentation to account for potential unobserved heterogeneity between observations. The mean speed was negatively and positively associated with the frequency of property damage-only (PDO) and severe accidents, respectively. Moreover, driver-related variables, including tailgating, distracted driving, and speeding, played key mediation roles in associating traffic and environmental factors with the crash risk. The higher the mean speed and the lower the traffic volume, the higher odds of distracted driving. Distracted driving was, in turn, associated with the higher vulnerable road users (VRU) accidents and single-vehicle accidents, triggering a higher frequency of severe accidents. Moreover, lower mean speed and higher traffic volume were positively correlated with the percentage of tailgating violations, which, in turn, predicted multi-vehicle accidents as the main predictor of PDO crash frequency. In conclusion, the mean speed effects on the crash risk are entirely different for each crash type through distinct crash mechanisms. Hence, the distinct distribution of crash types in different datasets might have led to current inconsistent results in the literature.

## 1. Introduction

In high-income countries, speed contributes to about 30% of deaths on the road, while in some low-income and middle-income countries (LMICs), speed is estimated to be the main contributory factor in about half of all road crashes [1]. Indeed, in LMICs, speeding-related enforcements and regulations are not effective enough compared with inhibitor costs of speeding offenses in high-income countries. While in many high-income countries, there is increasing use of built-in mechanisms in heavy vehicles to restrict speeds above a certain limit, such instruments are frequently resisted in LMICs for commercial reasons or else, if installed, are disabled by the operators [2]. Commercial operations are based often on timetables that pressure drivers to speed. In many LMICs, the pay of bus drivers is related to ticket receipts, which encourages high speeds. Moreover, road infrastructures in high-income countries are almost developed based on the forgiving design concept, which might reduce the occurrence and severity of accidents, even if the drivers violate the speed limit or commit other flaws while driving. Speeding is defined as simply violating posted speed limits or driving at unsafe speeds regarding the existent road-environmental conditions at a specific time or location (e.g., sharp curve or inclement weather). Indeed, the role of the operating traffic condition and highway features, termed road message cues, on the driver’s speed choice has been extensively investigated [3].

Whether it is adopting speeds deemed unsafe for the corresponding condition or violating the speed limit, speeding is a leading factor contributing to crash risk and severity [4]. The common sense and basic laws of physics imply that the higher the speed, the less time (perception-reaction time) the driver has to react to an issue that could suddenly arise on the road and, thus, the higher the risk of not taking appropriate action [5]. Moreover, empirical evidence suggests that higher average speeds increase the inattention risk [6]. Nonetheless, modeling this association is not as straightforward as it might seem.

The speed-crash relationship is almost confounded by various other influential factors (weather, roadway, traffic characteristics, etc.). As a result, the findings are inconsistent across studies with different data aggregations, modeling approaches, and speed measures (speed variation, mean speed, etc.) [7]. While it is well established in the literature that speed variation has a negative effect on traffic safety, the outcomes of previous research on the effects of mean speed on crash occurrence are counterintuitive. Some studies found a negative or insignificant association between mean speed and crash frequency [8–10]. Scholars suggest that confounding factors like weather characteristics would influence both the speed and crash likelihood and consequently conceal the true relationship [7]. The studies that indicated a negative speed-crash risk relationship have almost attributed their findings to the confounding factors masking the true association. Meanwhile, a limited number of studies have explicitly investigated the confounding effects. The key relevant work is presented in the following paragraphs.

Taylor et al. [11] investigated speed effects on the frequency of injury crashes on rural roads in the U.K. They first developed a Poisson model based on the complete dataset, illustrating a negative speed-crash relationship. Then, the authors separated the data into homogeneous groups based on the characteristics of the road that define its quality. The new results indicated a positive association within each group, which revealed the necessity for considering the potential unobserved heterogeneity between observations. Indeed, the road quality variable influences both the operating speed and crash likelihood, thus masking the true relationship. The higher quality of road design features would be associated with a higher mean speed and less crash risk. Hence, if the confounding variable is overlooked, the statistical analysis would conclude that “the higher the speed, the lower the crash risk”. Moreover, recent studies illustrated the significance of spatial correlations in causing heterogeneity in the distribution of speed and speed variation in a road network [12]. The heterogeneity that affects the relationship between speed and crashes might come from those unobserved effects. The findings suggest that these spatiotemporal dependencies cause crashes in temporal or spatial proximity to have similar severity outcomes [13]. Nonetheless, the current literature almost considers crash observations as independent events, which might result in biased parameter estimates and inferences.

Yu et al. [14] employed a Bayesian inference approach to model accidents using one year’s crash data on the I-70 in Colorado. The study utilized real-time weather, traffic, and road geometry variables and illustrated the significant contribution of weather attributes to crash occurrence. The results also indicated that the lower speed at the crash segment and the higher occupancy at the upstream segment 5–10 min prior to the crash time are associated with the higher crash risk. This might be attributed to the congestion effects. However, both the lower speed and the higher crash likelihood might be the results of inclement weather conditions, in which case the speed-safety association would be affected by a third confounding variable.

Gargoum and El-Basyouny [5] adopted path analysis to identify and control for the variables confounding the speed-safety relationship. They investigated the possible confounding effects of road, traffic, and environmental characteristics. The results indicated that traffic volume and segment length affect both the speed and crash frequency, revealing significant confounding effects. Meanwhile, average vehicle length, posted speed limit, the presence of bus stop, and the presence of shoulder were only indirectly related to crash frequency through mean speed. They found a positive association between mean speed and crash frequency. Meanwhile, the standard deviation of speed seemed to be inversely related to collisions (P-value = 0.088).

Speed variation is used to illustrate the inconsistency of vehicle speed along a segment. Despite a variety of speed measurements and research methods, crash frequency has consistently been found to rise as speed variation increases [4, 15]. Park et al. [7] employed path analysis to account for the potential confounding effects of roadway and traffic-related variables. The results suggested that larger variability in operating speed is indicative of reduced smoothness in traffic operations, which, in turn, results in an increased risk of accidents. The study also indicated the significant confounding effects of traffic volume, segment length, and the residential area on the association of mean speed deviation from the posted speed with the frequency of severe outcome accidents. If such confounding effects are overlooked, the estimated coefficient of the direct association of speed and crash risk would be illusory due to the omitted-variable bias effects.

Hutton et al. [16] investigated the speeds of individual drivers along 100 study segments in a naturalistic driving study to evaluate variations within individual trips as well as variance among drivers on the same road segment. The results illustrated that road features significantly impact the speed-safety relationship. Moreover, the findings suggest that speed variation between trips is related to multi-vehicle collisions. Other evaluated speed measures, such as space mean speed and mean operating speed, either had a negative or insignificant correlation with crash frequency.

**Fig 1:**
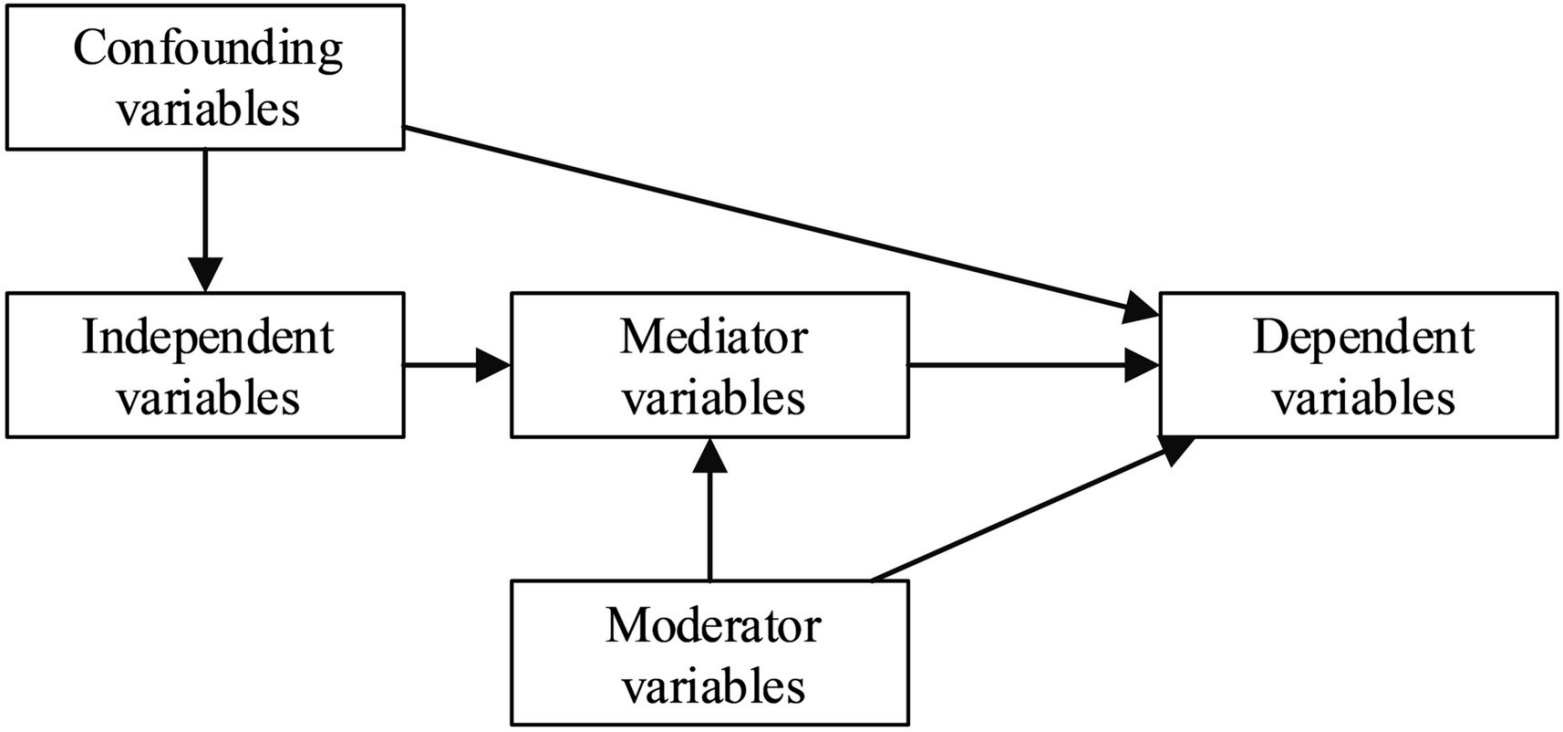
Four categories of variables influencing traffic accidents (Elvik et al. [17]).

Elvik et al. [17] proposed a causal diagram illustrating the complex effects of mediating, moderating, and confounding factors on the association of the dependent (DV) (crash frequency) and independent (speed) variable (IV) (see Fig 1). The mediation effects illustrate how the target variable is indirectly influenced by the explanatory variables. The mediators are risk factors that, if modified, would affect the negative outcome of the crash contributing factors. Accordingly, identifying such mediation effects not only enhances our understanding of the crash mechanism but also potentially assists policymakers in identifying risk factors for interventions. Additionally, if such variables are overlooked, the estimated direct effect between the DV and IV would be biased, termed omitted variable bias (endogeneity) [18]. The moderating variables are factors that, if considered, might influence the intensity of speed effects on collisions in the model. For example, the pavement condition has a moderation effect on the speed-crash interaction, where lower friction might boost the positive effect of speed on the crash likelihood [5].

From a statistical modeling point of view, the inconclusive results of the previous research would be attributable to endogeneity, multicollinearity among regressors, and inappropriate modeling frameworks [18]. The flexibility of the first-generation statistical techniques, which mainly involve regression-based approaches, is limited for modeling high-dimensional safety data. Indeed, the inherent collinearity between variables drives scholars to eliminate the number of variables to comply with the predictors’ independence assumption [19]. The ability of first-generation techniques is limited for tackling high-dimensional data. However, recent studies have improved these approaches by penalizing the collinear variables with ridge [20] or LASSO [21] shrinks and extend the use of regression in the road safety analysis. These studies indicated that consideration of multicollinearity among the variables would not only improve the models’ fit but also brings new insights into the effects of crash risk factors on traffic safety. Moreover, the classical methods have mainly attempted to build a direct relationship between various explanatory variables and crash frequency, even though some variables might indirectly influence the target variable through mediators. Besides, the classical methods cannot simultaneously contain various DVs, termed endogenous variables, like crash frequency by severity levels. Nonetheless, research has illustrated that IVs in crash modeling have unique effects on different crash types and severities [22]. Hence, such different effects might cancel each other and result in misleading conclusions.

For these reasons, exploring alternative statistical methods would allow uncovering the complex relationship between the risk factors and crash likelihood. Structural equation modeling (SEM) is recently employed in traffic safety studies to unravel such complex associations. However, this method has not yet sufficiently adapted to the unique features of traffic safety data. The current studies analyzed the full datasets, implicitly assuming that the data stem from homogenous observations. Nevertheless, unobserved heterogeneity might lead to biased estimates and misleading conclusions [23]. For example, when analyzing the aggregate data, positive and negative group-specific effects might cancel each other, triggering type II errors [24].

### 1.1 Research motivation and objectives

Previous studies have investigated the mediation effects of speed in the association of road features with crash occurrence [5]. Nevertheless, no study has analyzed the mediation effects that driver errors might have on the speed-safety association to suggest potential interventions. A recent study has used the random parameter structural equation modeling (SEM) approach to account for unobserved heterogeneity [25]. Nonetheless, this method cannot estimate group-specific coefficients for latent classes of data. Meanwhile, the group-specific coefficients would unravel the different crash mechanisms which might exist in each data segment. Hence, the current study aimed to introduce the FIMIX-PLS method to detect potential unobserved heterogeneity among observations, pushing forward the current methodological frontier of SEM applications in traffic safety. The other goal was to shed light on the mean-speed and crash risk relationship for different crash types/severities by accounting for the mediation effects of human factors and the intercorrelations between explanatory variables.

## 2. Literature review

### 2.1. The relationship between speed and crash frequency

The early studies stated that the speed-crash association could be described by a “U-shaped” curve, in which the deviations from the modus speed trigger accidents [26]. In contrast, a majority of other efforts indicated power [27], linear [11] or exponential [28] forms for the positive speed-crash relationship. Taylor et al. [11] investigated the speed-crash relationship for English rural single carriageway roads, which resulted in log-linear regression models. The research illustrated the masking effects that road quality measures impose on the speed-crash association, as the negative effect of mean speed on crash frequency was inversed after the road segments were grouped into homogenous sub-datasets. Nilsson [27] introduced the power model for the association of speed and road safety, which has become one of the dominant models for this association. Hauer [28] argued that an exponential function is a better model of the association of speed and road safety than the Power Model, mainly because the effect of a given relative change in speed does not depend on initial speed according to the Power Model, which seems implausible. While previous research analyzed the aggregate data, Kloeden et al. [29] best described the relationship between individual vehicle speed and crash rate, where an exponential relationship between the speed and the severe outcome crashes was found. Elvik [30], in a meta-analysis study, analyzed over 115 studies containing a total of 526 estimates of the relationship between changes in the mean speed of traffic and changes in the number of accidents or accident victims. The study concluded that the analyses presented lend stronger support to the use of an exponential function than to the use of a power model. A recent meta-analysis research concentrated on the new results in recent publications and evaluated the strength of traditional power and exponential models [31]. This study updated the estimates of the relationship between speed and road safety at the aggregate and individual levels, revealing that both the power model and the exponential model fit the data well, but the exponential model fits best. Moreover, the study indicated that the initial speed influences the exponents in the power model, where higher initial speeds are associated with a sharper increase in crash counts, even with a similar relative change in mean speed. Elvik et al. [31] argued that new safety systems on cars have made road safety outcomes more sensitive to changes in speed. Indeed, electronic stability control and emergency brake assistance systems have made crash avoidance possible with even a little decrease in vehicle speed.

Subsequent studies illustrated that speed variation is so influential on crash risk, which conceals the simultaneous effects of mean speed, proposing that “variance kills, not speed” [7, 32]. Quddus [32] stated that the insignificant association might be due to the study’s failure to account for segment characteristics like within- and between-lane speed differences. Choudhary et al. [33] aggregated crash records on a freeway section based on the similarities of the conditions just before their occurrence (e.g., condition-based approach) and modeled using Multivariate Poisson lognormal regression. Results indicated that crash rates increase as the within-lane speed variations raise, especially at higher traffic volumes. Higher speeds coupled with greater volume and high between-lanes speed variation also increase the crash likelihood. Another cross-sectional study on Hong Kong freeway segments utilized GPS data of 480 taxis to investigate the speed-safety association. The results indicated that mean speed is inversely related to crash frequency. The authors inferred that higher mean speed was associated with shorter time exposure; thus, crash frequencies decreased [34]. This study was among the rare studies that found no significant relation between speed variation and crash occurrence. Moreover, some studies stipulated that both speed variations and mean speed positively correlate with the crash likelihood [15, 35].

Wang et al. [15] investigated the association between speed and crash risk in eight arterials in Shanghai. The result indicated that a 1% increase in mean operating speed was associated with a 0.70% increase in total crash counts. Also, various other studies found a positive relationship between mean speed and crash frequency [5, 36]. Meanwhile, the results of some studies contradicted this view, reporting negative [8, 9] or insignificant effects [10]. Moreover, recent real-time crash prediction studies have reported a negative relationship between mean speed and crashes, while speed variation was positively related to crash frequency [37, 38].

Numerous studies evaluated the speed limit impacts on crash trends in a before-after study framework. Elvik [39] investigated 115 such studies whose results generally indicated a decline in crash counts when the speed limit was reduced. In the fixed-effect statistical weight, only about 5.7% of studies found unfavorable outcomes for traffic safety. These exceptional findings have almost been attributed to the most important confounding factors in the before-after research design, such as common trend, effects of chance, and the regression to the mean (RTM) phenomenon. To handle such effects, using the empirical bays (EB) method and defining comparison groups are common modifications that have been imposed on classic before-after study design [40].

Recent studies criticized the previous research for their link-based data aggregation, stating that those studies might suffer from aggregation bias [41]. They suggested that the speed-crash relationship completely differs for different crash types, and this unobserved heterogeneity might conceal the true association. Hence, they proposed scenario-based aggregation where similar pre-crash characteristics are considered for aggregation in a multivariate count modeling framework [33].

Hauer [28] criticized the previous studies as they failed to illustrate whether the observed differences in relative accident rate are due to the probability of accident involvement or due to the severity of the outcome. The author argued that due to the “frequency-severity indeterminacy”, the current results are inconclusive and highly affected by the distribution of crash types and severities. Scholars also stated that the current contradictory results might be attributed to the fact that most studies neglect the crucial role of mediation effects [42].

The previous studies that found a negative speed-crash relationship have almost related their results to potential unobserved heterogeneity, confounding factors, and endogeneity. Nonetheless, they did not investigate the underlying mechanisms, such as mediation effects and the proportion of different crash types, which might explain the results.

### 2.2. SEM applications in accident analysis

Studies have utilized SEM to incorporate the crash severity-related features like the number of injured and dead individuals, as well as the number of vehicles involved, into a latent construct termed “crash size” to have a comprehensive and more reliable measure of actual crash severity. Moreover, the crash contributing factors have been integrated into unmeasurable variables, namely, driver, road, and vehicle, which, in turn, allows researchers to compare the relative contribution of each latent variable in the crash size [43]. Besides, scholars introduced the “crash risk” latent variable by integrating the surrogate safety measures and crash data to model specific crash types with low frequency [25]. The capability of SEM for modeling indirect effects has also attracted the attention of naturalistic driving studies [44] and real-time crash prediction models [45] to account for the complex associations between intercorrelated variables.

Previous studies also tried to adopt SEM to unique features of safety data and overcome methodological shortcomings. Hyun et al. [46] introduced generalized SEM (GSEM) to combine the power and flexibility of both SEM and GLM methods. Standard SEM assumes that the endogenous variable is normally and non-continuously distributed [47]. A Generalized Structural Equation Model (GSEM) overcomes this limitation by allowing the model to possess both continuous and discrete variables grouped together in the same latent construct [46]. Gharraie and Sacchi [48] investigated the severity of wildlife-vehicle crashes in Canada, utilizing structural equation modeling (SEM) with generalized (ordered probit) links. Generalized SEM with discrete response links was in compliance with the discrete and ordinal nature of the response variable. In contrast to the previous work that adopted the frequentist approach for developing SEM-based crash prediction models, Kim et al. [49] developed SEMs in the Bayesian framework to investigate crash severity in relation to various variables, including accessibility, vehicle, human, and roadway-related features. Because of the flexibility of modeling both continuous and discrete variables in the Bayesian approach, the latent variable “crash” was constructed by combining ordinal (the extent of damage to vehicles and injury types) and continuous random variables (the number of vehicles involved) together in their study. Yang et al. [25] developed a new SEM with conditional autoregressive spatial effect and corridor-level random parameters (SEM-CAR-RP) to account both for the spatial autocorrelation and unobserved heterogeneity in crash frequency modeling. Although random parameter SEM has a significant advantage over its standard counterpart regarding the overall fit quality, it does not allow researchers to investigate the source of heterogeneity.

Although previous studies tried to relax the restrictive distributional assumptions in standard SEM, SEM is not well adapted to the heterogeneity between observations as one of the unique features of crash data. Some studies used multigroup analysis to estimate group-specific coefficients for predefined specific data segments based on a-priori information [50]. Also, scholars have adopted K-means clustering in a sequential data preprocessing procedure to account for unobserved heterogeneity [45]. Meanwhile, none of these methods is considered satisfactory, as observable characteristics often gloss over the true source of heterogeneity [51]. In line with this statement, scholars criticized the study of Taylor et al. [11] since the data segmentation in that study was based on variables considered explanatory variables in the model [52]. Besides, the clustering methods cannot unravel heterogeneity concerning the structural model, and they also suffer from conceptual shortcomings [51]. Hence, social scientists devoted considerable efforts to developing model-based segmentation techniques. Consequently, this study contributes to the current methodology by introducing FIMIX-PLS segmentation [53] as a model-based approach that addresses the mentioned shortcomings.

## 3. Data description and hypotheses

**Fig 2:**
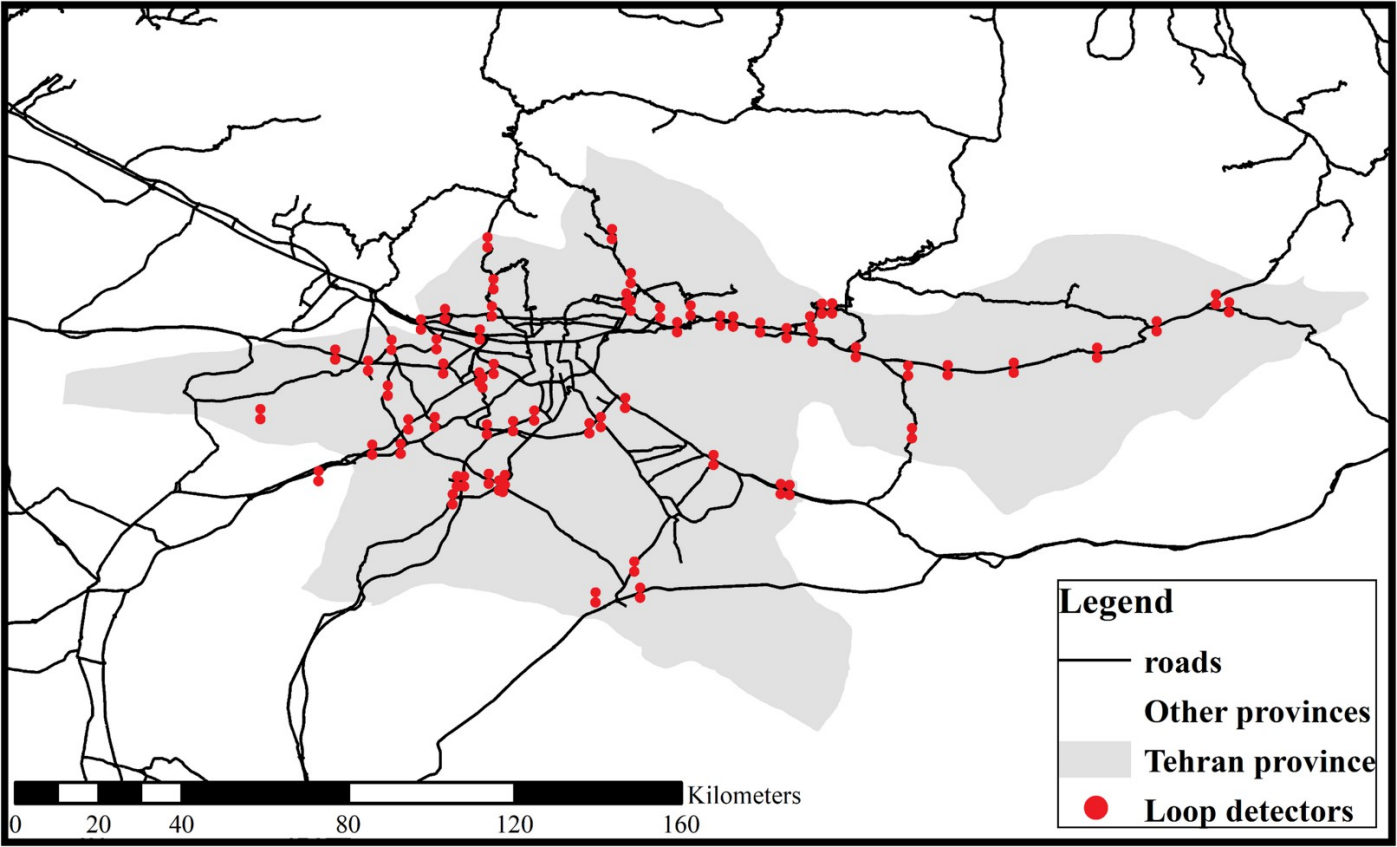
Map of rural highways and loop detectors in Tehran province. Reprinted from [54] under a CC BY license, with permission from the Iran Ministry of Roads and Urban Development (MRUD), original copyright 2023.

This study utilized the surveillance data of road traffic crashes on rural multilane highways of Tehran, Iran, developed by the National Traffic Police (NAJA). Traffic police collect all traffic crashes by filling out the standard “COM114” forms (on which information about crashes and their causes is recorded). The dataset includes variables such as road condition, crash severity, crash type, and crash reason. Since 2020, the Ministry of Roads and Urban Development (MRUD) has gathered police reports and the loop detectors’ data. One hundred six loop detectors across all rural multilane highways of Tehran province record the time-mean speed (TMS) of vehicles, the number of tailgating and speeding vehicles, and the traffic volume. The study region and the map of multilane highways and loop detectors are illustrated in Fig 2. As the exact location of accidents has not been recorded, it was not feasible to link each crash with its corresponding traffic condition. Hence, daily accident data along with daily traffic characteristics across the study area was examined to discover underlying crash patterns, covering a two-year period, 2020–2021. Descriptive statistics of daily crash records in the study region are detailed in Table 1.

**Table 1:** Descriptive statistics of daily crash records in the study region.

Crash severity/type	Number of crash records per day [mean (SD)]	Share of total daily accidents [mean (SD)]
***Multi-vehicle accidents***	18.66(10.10)	0.63(0.19)
***Single-vehicle accidents***	5.38(3.62)	0.19(0.10)
***vulnerable road users (pedestrians & motorcyclist) accidents***	3.60(2.96)	0.12(0.09)
***Fatal and injury accidents***	8.98(4.58)	0.27(0.09)
***Property damage only accidents***	23.46(9.72)	0.73(0.09)

Note: Share of total daily accidents [= Number of crash records per day (for a given crash type or crash severity level) / Number of total daily crashes].

### 3.1. Tailgating

Tailgating is defined as following a vehicle with an unsafe time headway of less than two seconds [55]. This buffer gives at least a minimum time equivalent to the perception reaction time (PRT) to the following driver to avoid a collision with the front vehicle [56]. Research has shown that tailgating is the main predictor of multi-vehicle accidents, especially rear-end collisions [57, 58]. Moreover, studies indicated that tailgating is more redundant on congested roads [59]. Accordingly, this study hypothesizes:

H1. The higher the traffic flow, the higher the percentage of tailgaters on the road; thus, the higher the likelihood of multi-vehicle accidents.

This research employed the loop detector data for identifying the daily percentage of tailgaters relative to all detected vehicles, considering a minimum bumper-to-bumper headway of two seconds. Several attributes were examined as potential explanatory variables; the selection of the predictors was according to their perceived influence on speed and crashes. This was mainly distinguished by investigating the findings of previous studies. Descriptive statistics of the selected variables are detailed in Table 2.

**Table 2:** Descriptive statistics of variables.

Variable	Abbreviation	Range	unit	Mean (SD)
***Human factors***				
***Tailgating***	***Tailgating***	0–1	DL	0.20(0.03)
***Speeding***	***Speeding***	0–1	DL	0.08(0.07)
***Distracted driving***	***Distraction***	0–1	DL	0.10(0.08)
***Traffic & environmental characteristics***				
***Traffic flow***	***TrafficFlow***	532,859–3,262,946	Vehicles	2,046,593(425227)
***Mean speed***	***MeanSpeed***	70.3–82.5	Kmhr	78.57(1.22)
***Wet pavement***	***WetPav***	0–1	DL	0.05(0.14)
***Crash type***				
***Proportion of Multi-vehicle accidents***	***MultiVeh***	0–1	DL	0.63(0.19)
***Proportion of Single-vehicle accidents***	***SingleVeh***	0–1	DL	0.19(0.10)
***Proportion of vulnerable road users*** ***(pedestrians & motorcyclist) accidents***	***VRU***	0–1	DL	0.12(0.09)
***Crash Frequency***				
***Fatal and injury accidents frequency***	***FI***	0–22	numbers	8.98(4.58)
***Property damage only accidents frequency***	***PDO***	0–48	numbers	23.46(9.72)

Note: DL (Dimensionless quantity or Ratio).

### 3.2. Speeding

Speeding is one of the main contributing factors to accident risk and severe crash outcomes [4]. Taylor et al. [11] found that if the percentage of vehicles exceeding the speed limit is reduced from 20% to zero, accidents would be roughly halved on U.K. rural roads. Moreover, previous studies found that perceptual and road message cues play a determinative role in speeding behavior [60]. The findings illustrate that individuals drive faster than they perceive after adopting highway speeds and entering a lower-level facility, leading to speed violations [61]. Moreover, the speed violation of adjacent vehicles has been shown to push drivers toward speeding [62]; this study hypothesizes:

H2. The higher the average speed, the higher the percentage of speed violations on the road; thus, the higher the likelihood of accidents with different crash types and severities.

The loop detectors record the number of vehicles that violate the posted speed. This information was used to measure the daily percentage of speed violators on the road.

### 3.3. Distracted driving

The research illustrated that demanding situations, namely, inclement weather [63], urban environment [64], and unfamiliarity with the road [65], have suppressor effects on distracted driving. A naturalistic driving study indicated that distractions were more likely under clear weather conditions and low traffic congestion [66]. Scholars termed this effect as risk compensation, where the higher driver’s workload in complex traffic situations triggers a decrease in subjective safety and, subsequently an increase in self-regulating alertness [67].

Distracted driving is a contributing factor in about 80% of accidents and 65% of near-crashes in the U.S. [68]. Moreover, driver’s distraction is one of the main predictors of various crash types, including vulnerable road user (VRU) [69], rollover [70], run-off-the-road [71], and multi-vehicle accidents [72]. Hence, this study hypothesizes:

H3. The lower the traffic volume and the higher the mean speed, the higher the prevalence of distracted driving; thus, the higher the likelihood of different crash types, which, in turn, are related to the frequency of specific crash severity levels.

Police report the presence of distracted driving as one of the crash reasons, which is recorded along with other crash information. The police fill out a standard checklist (COM114 form) at the crash scene. The crash report has a section for human factors contributing significantly to crash occurrence. Police officers are trained to distinguish between human factors based on instructions. The most important human factors, like fatigue, drunk driving, and distracted driving, have a distinct reporting field in the form. The police officers will take self-reports from the parties involved (if survived) and non-involved witnesses (e.g., vehicle passengers or pedestrians). These side reports will then be attached in the narrative section at the end of the form. Moreover, some field measurements are conducted, including checking the length of brake slips, the location of the first impact, and also the manner of the collision on the road based on the guidelines. Finally, police label the accident as distraction-related based on the definition. Distraction from driving occurs when drivers divert their attention from the driving task to some other activity that can be inside the motor vehicle or outside the motor vehicle. Driving while daydreaming (cognitive distraction) is also identified as distracted driving. Physical impairments (alcohol, disabilities related to age, etc.) or psychological conditions (anger, depression, etc.) are not identified as distractions but are located in the “Other human factors” field. This study adopted the proportion of distraction-related accidents to total daily crashes to measure the prevalence of distracted driving on the road.

### 3.4. Traffic and environmental characteristics

The basic knowledge of traffic flow theory implies that the mean speed would be lower during higher traffic volumes. Moreover, traffic flow as a measure of exposure has almost been the main predictor of accidents [5]. Besides, it is found to be the main factor that confounds the association between speed and safety [4]. This paper used the total traffic flow per day to measure traffic exposure. Moreover, the daily mean speed of vehicles was also employed from the loop detector data.

Inclement weather and wet pavement are shown to be associated with lower mean speeds and traffic volumes, which can be attributable to driver’s risk compensation and a decline in unnecessary travel [73]. Moreover, wet pavement condition is found to be associated with different crash severity and types, especially multi-vehicle [74] and run-off-the-road [75] accidents. Moreover, the previous studies highlighted the potential confounding effects of weather on the speed-safety interaction [17]. The percentage of accidents on wet pavements is used as the measure of environmental status in this research. Hence, this study hypothesizes:

H4. Traffic volume is associated with both the frequency of accidents by different crash types/severities and with the mean speed.
H5. The wet pavement condition is associated both with traffic characteristics (e.g., traffic volume, mean speed) and with different crash types/severities.

### 3.5. Conceptual model

**Fig 3:**
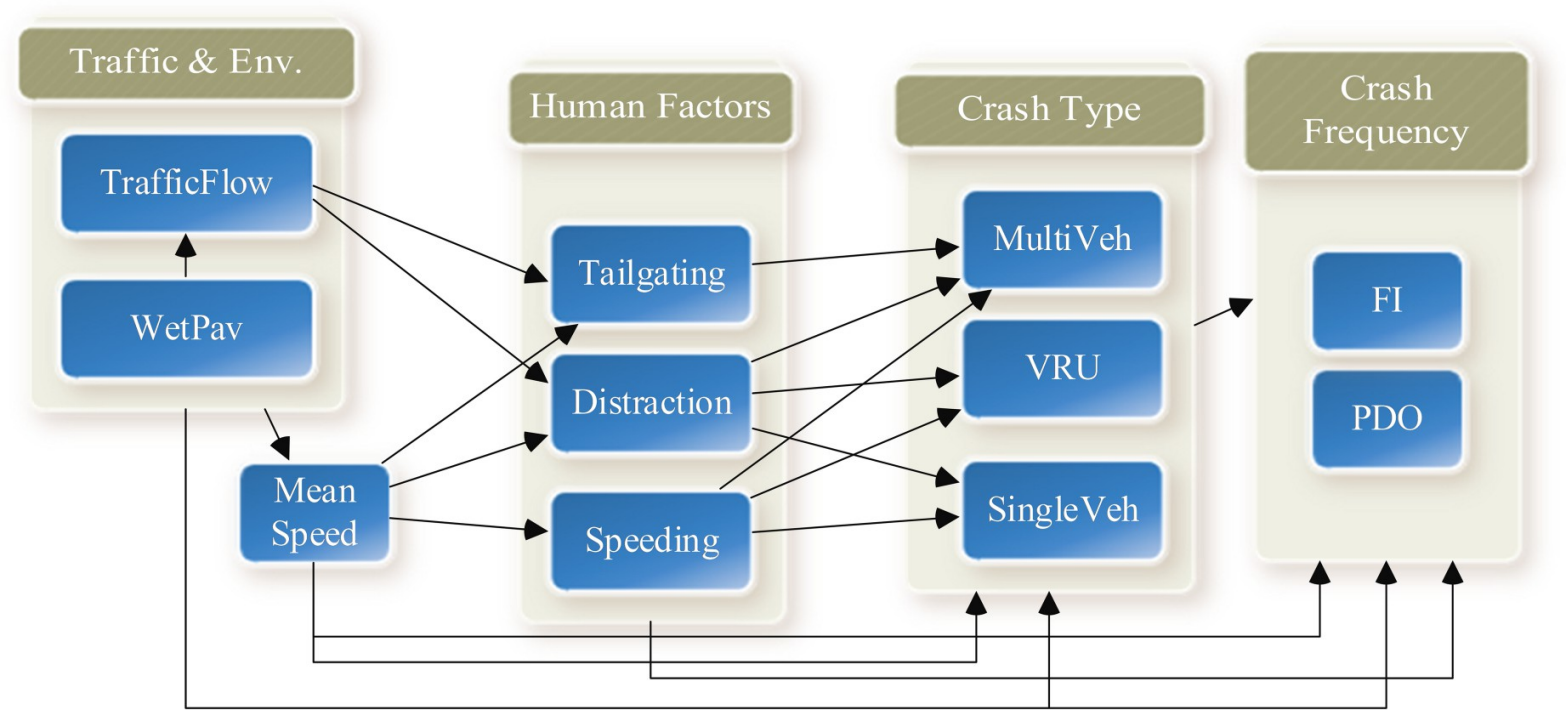
Conceptual model.

The emergence of SEM applications in social sciences led to a revolution in the methodology of causal inference. Nevertheless, researchers should first draw a conceptual structure based on the domain knowledge to support the causal inference from the model. Then, the structural model is evaluated regarding the extent to which it explains the data [76]. The conceptual model is depicted in Fig 3.

## 4. Methodology

This study adopted PLS-PM for crash causal analysis, a form of PLS-SEM, where all the variables are measurable (i.e., manifest variables). The SEM method was employed for two reasons. First, SEM enables the researchers to unravel the mediation effects, which, in turn, brings valuable insights into the underlying crash mechanism and assists policymakers with developing effective countermeasures. Second, as proposed by Elvik et al. [17], understanding the true speed-safety association would not be possible without considering the complex moderating, confounding, and mediating effects, which is not possible in the first-generation techniques.

### 4.1. Partial least square path modeling (PLS-PM)

There are two types of SEMs, as the second-generation technics, namely Partial Least Squares SEM (PLS-SEM) and Covariance-Based SEM (CB-SEM). CB-SEM is useful mainly in confirmatory studies when research aims to examine a theory in the field. In contrast, PLS-SEM approach is exploratory in nature. It is mainly used when there is not a well-established theory or the prediction of endogenous variables has the main priority [77]; because the PLS-SEM estimates the structural model so that a higher proportion of variance in the endogenous variables is explained for. On the other hand, CB-SEM minimizes the differences between the estimated and observed covariance matrices to estimate the model parameters. While CB-SEM seeks to gain the best fit for the data, PLS-SEM mainly focuses on best predicting the endogenous variables [78]. Accordingly, the underlying assumptions (e.g., required sample size and statistical distribution) and the implications are different in the two approaches [78].

PLS-SEM is labeled as ‘‘Soft modeling” since it does not contain strict assumptions about normal data distribution, measurement scales, and sample size, as in CB-SEM [77]. The significance of the relationships is evaluated utilizing a non-parametric bootstrapping test through resampling with replacement from the original sample [77]. The P-values provided by bootstrapping procedure indicate the stability of the estimated path coefficients [78]. When applying PLS-SEM, scholars also benefit from high efficiency in parameter estimation, which is reflected in the method’s greater statistical power than CB-SEM. Greater statistical power means that PLS-SEM is more likely to render a specific association significant when it is actually significant in the population [77].

PLS-SEM was chosen over its covariance-based counterpart for three reasons. First, the main aim of this research was to predict target variables, especially crash frequency by severity, and there is not a well-established theory for the whole model. Second, the structural model would be very complex, containing various hypotheses, while the sample size is relatively small (see Fig 3). It is worth noting that the complexity of the PLS-PM model does not significantly increase Type I and Type II errors since distinct regression analyses are used to estimate the paths related to each block [79]. Moreover, PLS-SEM is preferred over CB-SEM because its estimation procedure is guaranteed to converge to the optimum solution after a few iterations. Nonetheless, that is not the case for CB-SEM, which relies on maximum likelihood estimation [77]. Indeed, the identification issues in the covariance-based approach limit its application when the underlying structural model is complex [77]. Third, the multivariate normality assumption in the covariance-based approach with maximum likelihood estimation is restrictive, especially when analyzing crash data, which, if violated, would result in biased estimates [80]. The S2 File illustrates the normality test results conducted on the datasets used in this research. The results of the Kolmogorov-Smirnov test are used to test the null hypothesis that the set of data comes from a normal distribution. It is evident that the data is slightly skewed to the right. Empirical findings confirm that the PLS-PM’s statistical properties provide very robust model estimations with data that have normal as well as extremely nonnormal (i.e., skewness and/or kurtosis) distributional properties [81]. Hence, the PLS-PM model would be a proper modeling framework for this study. Despite the flexibility of PLS-SEM in crash causal analysis, only a few studies have recently adopted this method on safety data [80, 82]. The details of PLS-PM method are proposed in S1 Appendix.

Smart-PLS software, version 3.2.8, was used to perform path analysis, accounting for direct and indirect effects. Before model estimation, the software standardizes the input data. Standardization is done by subtracting the variable’s mean from each observation and dividing the result by the variable’s standard error. The analysis was implemented using 1000 bootstrapping iterations to examine the statistical significance of the hypothesized associations. The overall fit of the structural model was assessed using Normed Fit Index (NFI), Standardized Root Mean Square Residual (SRMR), and chi-square statistics. The satisfactory level (NFI≥0.9) of the NFI criterion was adopted as the cut-off point for the model’s overall fit [83]. Additionally, the cut-off point of 0.08 was employed as the maximum acceptable SRMR value [84]. The chi-square (*χ*^2^) statistic evaluates the magnitude of discrepancy between the fitted and sample covariance matrices [84]. As the *χ*^2^ test is susceptible to sample size, the chi-square divided by the degrees of freedom (*χ*^2^/*df*) criterion was employed as the model fit index, with values under five interpreted as acceptable [85]. Besides, the model’s prediction power was examined using the predictive relevance index (*Q*^2^) [86], in which a blindfolding procedure is used for cross-validation. The *Q*^2^ value above zero indicates the path model’s predictive relevance for a dependent variable [77].

### 4.2. Mediation effect analysis

Mediation effect analysis illustrates how a variable x transits its effects to the endogenous variable y. Indeed, mediation is employed to examine whether the effect of x on y is (i) indirect only (through a mediator variable, *m*), (ii) direct only, or (iii) both direct and indirect. Case (iii) is known as partial mediation, whereas case (i) is termed full mediation [5].

A simple mediation represents a model in which *x* influences y through a single mediator variable, as is depicted in Fig 1. Eqs (1) and (2) are linear regressions that indicate a simple mediation model. Eq (1) indicates the combination of paths from *x* to y and m to y, while Eq (2) represents the path from *x* to *m* [5].

$$y_{i}=\beta_{0}+\beta_{1}m_{i}+\beta_{2}x_{i}+\varepsilon_{1i}$$ (1)

$$m_{i}=\gamma_{0}+\gamma_{1}x_{i}+\varepsilon_{2i}$$ (2)

where, *y*_*i*_ indicates the target variable; *x*_*i*_ denotes the exogenous variables (IVs); *m*_*i*_ is the mediator variable; *ε*_1*i*_ and *ε*_2*i*_ are the error terms; *β*_0_ and *γ*_0_ are the intercepts; and *β*_1,_*β*_2_ and *γ*_1_ are the path coefficients where their values denote the magnitude of change in their corresponding endogenous variables (i.e., *y*_*i*_, *m*_*i*_) in response to a unit change in their related explanatory variables. *β*_2_ denotes the direct effect of x on y, whereas, the product-of-coefficients estimator (*γ*_1_*β*_1_) estimates the indirect effect of *x* on *y*.

If the inclusion of mediating variable results in a statistically insignificant direct effect, this would be inferred as a full mediation effect [87]. The Variance Accounted for (VAF) criterion was used to evaluate the extent of mediation effects in other cases. The VAF indicates the proportion of the target variable’s (y) variance, explained by the mediation effects to the variance accounted for through total effects (e.g., direct and indirect). The VAF values between 0.2 and 0.8 denote partial mediation, while values higher than 0.8 indicate full mediation [77]. The Smart-PLS software, version 3.2.8, reports the values of total direct and indirect effects between two variables in the structural model, as well as their statistical significance.

### 4.3. Finite mixture partial least squares (FIMIX-PLS) segmentation

Relying on the mixture regression concept, FIMIX-PLS simultaneously estimates segment-specific path coefficients for a predefined number of segments and derives each observation’s segment membership [88]. Simulation studies indicated that FIMIX-PLS segmentation reveals the existence of heterogeneity in the path models reliably and accurately unravels the appropriate number of segments to retain from the data [51]. The details of the FIMIX-PLS method are proposed in S2 Appendix. Finite mixture partial least squares (FIMIX-PLS) is a latent class segmentation method.

The optimum number of segments (K) is derived by iteration, starting from a one-segment scenario, in which the data is thought to be entirely homogenous. FIMIX-PLS is run iteratively with a higher K value, and the model’s fit is compared with the previous scenario using information criteria (e.g., BIC, Modified AIC 3 (AIC3), Consistent AIC (CAIC)). Finally, the best-fit scenario is evaluated for potential overfitting by entropy (EN) statistic, taking values between 0 and 1 [89]. If the EN value exceeds the cut-off point of 0.9, the following best-fit scenario will be checked until the optimum number of segments is achieved [24].

## 5. Results and discussion

**Fig 4:**
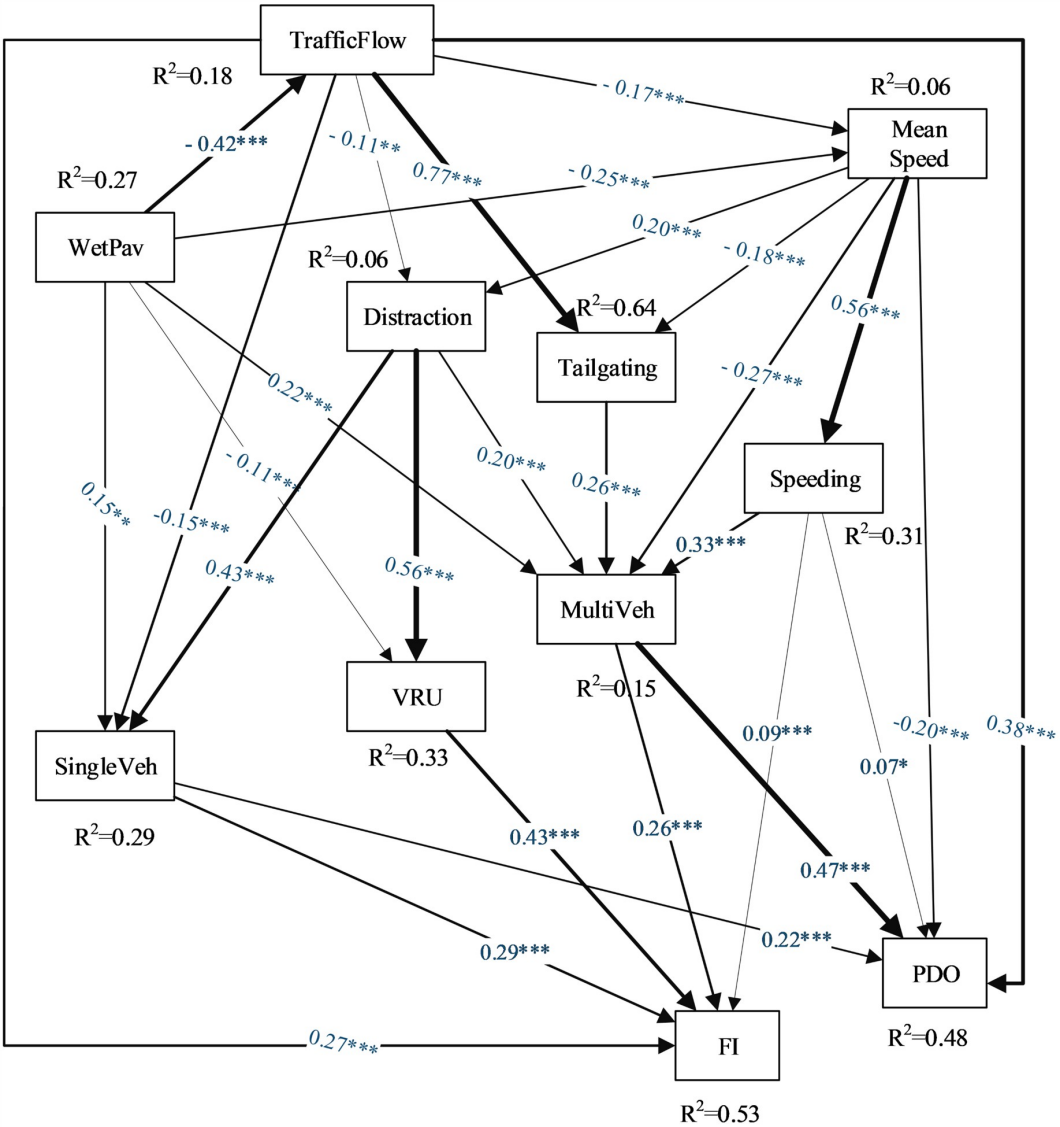
PLS path model results. Note: Values on vectors show standardized path coefficients. FI (Fatal and injury accidents frequency), PDO (Property damage only accidents frequency), SingleVeh (Proportion of Single-vehicle accidents), VRU (Proportion of vulnerable road user involved accidents), MultiVeh (Proportion of multi-vehicle accidents), WetPav (Wet pavement).* p < .05, ** p < .01, ***p < .001.

The hypothesized path model (see Fig 3) was considered the base structural model. Statistically insignificant paths were dropped sequentially by running multiple models. The final path diagram is depicted in Fig 4. The results indicate that the model explains 53%, 48%, 33%, 27%, and 15% of the variation in the severe crash frequency, PDO crash frequency, VRU accidents, Single-vehicle accidents (e.g., run-off-the-road, rollover, and fixed object accidents), and multi-vehicle accidents (e.g., rear-end, sideswipe, etc.), respectively. In Fig 4, the values on vectors represent the path coefficients, meaning that the greater the path coefficient values, the greater the effect of the explanatory variable on the endogenous variable.

Regarding the goodness of fit of the overall model (see Table 3), the NFI value represents a satisfactory model fit. Besides, the SRMR index and *χ*^2^/df statistics indicate an acceptable model fit. Meanwhile, it is recommended that instead of assessing goodness-of-fit, the structural model be evaluated based on out-of-sample predictive power [86]. As the predictive relevance value (*Q*^2^) was above 0.04 in all endogenous constructs, the above zero criterion was met (see Table 3).

**Table 3:** The cross-validated predictive relevance and overall fit indices of the PLS path model.

Endogenous variable	Predictive relevance	Overall fit indices		
	Q^2^	NFI	SRMR	*χ*^2^/*df*
***MeanSpeed***	0.04			
***TrafficFlow***	0.18			
***Distraction***	0.04			
***Speeding***	0.29			
***Tailgating***	0.62	0.90	0.07	2.56
***MultiVeh***	0.13			
***SingleVeh***	0.26			
***VRU***	0.32			
***FI***	0.51			
***PDO***	0.46			

Note: FI (Fatal and injury accidents), PDO (Property damage only accidents), VRU (Proportion of vulnerable road user involved accidents), SingleVeh (Proportion of Single-vehicle accidents), MultiVeh (Proportion of multi-vehicle accidents), WetPav (Wet pavement).

### 5.1. The explanatory variables’ contribution in different crash types

Overall, the path model outcomes shown in Table 4 seem reasonable and illustrate both direct and indirect effects of explanatory variables on the likelihood of crash occurrence by type. The inclusion of distracted driving in the association of traffic-related variables and VRU accidents resulted in insignificant direct relationships, implying a full mediation effect [87]. Hence, higher mean speed and lower traffic flow are indirectly associated with a higher proportion of VRU accidents through an increased tendency to drive distracted. This can be attributed to the risk compensation effect. The drivers are generally more cautious when the freedom of maneuver is restricted on congested roads, decreasing the level of subjective safety they perceive. Besides, driver distraction has been shown to be a main contributing factor in VRU accidents [90]. The results of the current study are in line with previous findings, as the path coefficients imply that a 10% increase in the prevalence of distraction-related collisions would be associated with a 5.7% increase in the proportion of VRU accidents. The association between distracted driving and VRU accidents is also by far stronger than the effects of the environment and traffic-related features. Conversely, the wet pavement condition was negatively associated with VRU accidents which might be explained by the mediating role that exposure could play in this relationship. In fact, the activity of vulnerable users on rainy days is minimized, and as a result, the odds of accident involvement for this group of road users are reduced.

**Table 4:** Direct, indirect, and total effects of explanatory variables on different crash types.

Crash type	explanatory variable	Direct effect	P-value	Total Indirect effects	P-value	Total effect	P-value	VAF
***VRU***	***MeanSpeed***	***-***	***-***	0.11	<0.001	0.11	<0.001	1
	***TrafficFlow***	-	-	-0.08	0.002	-0.08	0.002	1
	***WetPav***	-0.11	0.001	-	***-***	-0.11	0.004	-
	***Distraction***	0.57	<0.001	-	-	0.57	<0.001	-
***SingleVeh***	***MeanSpeed***	-	***-***	0.09	<0.001	0.09	<0.001	1
	***TrafficFlow***	-0.15	<0.001	-0.06	0.002	-0.22	<0.001	0.27
	***WetPav***	0.15	0.001	0.07	0.001	0.22	<0.001	0.32
	***Distraction***	0.43	<0.001	-	***-***	0.43	<0.001	-
***MultiVeh***	***MeanSpeed***	-0.27	<0.001	0.18	<0.001	-0.09	0.003	-2
	***TrafficFlow***	-	***-***	0.20	<0.001	0.20	<0.001	1
	***WetPav***	0.20	<0.001	-0.06	0.003	0.16	<0.001	0.38
	***Distraction***	0.20	<0.001	-	***-***	0.20	<0.001	-
	***Speeding***	0.33	<0.001	-	-	0.33	<0.001	-
	***Tailgating***	0.26	<0.001	-	***-***	0.26	<0.001	-

Note: VRU (Proportion of vulnerable road user involved accidents), SingleVeh (Proportion of Single-vehicle accidents),

MultiVeh (Proportion of multi-vehicle accidents), WetPav (Wet pavement), VAF (Variance Accounted For).

Reviewing Table 4, distracted driving and wet pavement condition are the main predictors of single-vehicle accidents. Traffic flow is negatively associated with single-vehicle accidents directly and indirectly through distracted driving, illustrating a partial mediation effect (0.2 <VAF<0.8). Moreover, the positive association between mean speed and single-vehicle accidents is fully mediated through distracted driving (VAF = 1). The path coefficients imply that a 10% increase in the prevalence of distraction-related collisions would be associated with a 4.3% increase in the proportion of single-vehicle accidents. The association between distracted driving and single-vehicle accidents is also by far stronger than the effects of the environment and traffic-related features. The path model also indicates that the wet pavement condition is negatively associated with the mean speed. Analogously, these findings would be inferred based on the risk compensation effect. Conclusively, the higher the mean speed and the lower traffic flow on the road, the higher the prevalence of distracted driving, thus, the higher likelihood of single-vehicle accidents. Moreover, wet pavement condition is positively associated with single-vehicle accidents. In line with this finding, research has shown that wet pavement increases the odds of run-off-the-road [75] and rollover accidents [91] due to the lower pavement friction. Moreover, wet pavement was negatively related to traffic flow, which, in turn, is inversely associated with single-vehicle accidents. Hence, traffic flow partially mediates the positive association between wet pavement and single-vehicle crashes. Indeed, inclement weather results in decline in unnecessary intercity travel and traffic flow. This, in turn, increases the prevalence of drivers’ distraction due to the perceived higher subjective safety. Consequently, the higher prevalence of distracted driving results in a higher likelihood of run-off-the-road, fixed object, and rollover accidents.

The total effects derived from the path model imply that speeding, tailgating, and distracted driving are the main contributing factors in multi-vehicle accidents, respectively (see Table 4). The results indicated that a 10% increase in the prevalence of speeding and tailgating violations on the road would be associated with a 3.3% and 2.6% increase in the share of multi-vehicle accidents from daily crash counts, respectively. The mean speed is inversely related to multi-vehicle accidents directly. In contrast, it is positively associated with multi-vehicle accidents by increasing the prevalence of distracted driving and speeding behavior. Accordingly, the total indirect effects of mean speed on multi-vehicle accidents contradict the direct association, evoking a suppressor full mediation effect manifested in the negative VAF value [77]. It is worth noting that if the mediation effects of human factors in this association had been overlooked, the estimated negative effect of mean speed on multi-vehicle accidents would have been overestimated, referred to as endogeneity bias [18]. The positive association between mean speed and speeding can be inferred based on the speed adaptation effect. Previous studies showed that drivers almost adapt to highway speeds and do not reduce speed when entering a lower-level facility [61]. Such behavior is predictable since the rural highways of Tehran province alternately pass through the suburban and industrial areas with lower speed limits.

The model also indicated that the higher the mean speed and the lower the traffic flow on the road, the lower tailgating violations, thus, the lower the multi-vehicle accidents. Indeed, the path coefficients suggest that a 10 kph increase in mean speed is associated with a 1.8% decrease in the prevalence of tailgating on the road, followed by a 1% decline in the share of multi-vehicle accidents. This, in turn, explains the reason behind the negative association between mean speed and multi-vehicle accidents partially. Moreover, wet pavement was positively associated with multi-vehicle accidents, although a moderate suppressor effect was found by some mediators. Indeed, the mediation effects imply that wet pavement reduces the highway’s mean speed, decreasing both speeding and distracted driving as the main predictors of multi-vehicle accidents. Meanwhile, the total effects are in line with previous studies, indicating that the lower pavement friction in adverse weather conditions, rises the probability of rear-end, sideswipe, and other types of multi-vehicle accidents [46, 92].

These findings might interpret the negative speed-crash association repeatedly reported in previous studies. The descriptive statistics in Table 2 indicate that, on average, 63% of daily accidents are of multi-vehicle type, which is five times more compared to the other two types of accidents. Meanwhile, the results indicate that the mean speed is positively associated with single-vehicle and VRU accidents while inversely related to multi-vehicle accidents. Hence, as the effects of mean speed on different crash types differ, the crash risk should be studied separately for each type of accident. Otherwise, the most frequent type of accidents in the study region would overwhelm the dissimilar effects of speed on other crash types. This, in turn, results in both a negative relationship between mean speed and crash likelihood and misleading interpretations. Although the frequency of single-vehicle and VRU accidents is relatively small, the results illustrate that they are responsible for a high proportion of accidents with severe outcomes (see Table 5).

**Table 5:** Direct, indirect, and total effects of explanatory variables on crash frequency by severity.

Crash frequency	explanatory variable	Direct effect	P-value	Total Indirect effect(s)	P-value	Total effect	P-value	VAF
	***MeanSpeed***	-0.20	<0.001	0.01	0.631	-0.19	<0.001	-
	***TrafficFlow***	0.38	<0.001	0.07	0.003	0.45	<0.001	0.16
	***Distraction***	-	-	0.19	<0.001	0.19	<0.001	1
***PDO***	***Speeding***	0.07	0.036	0.15	<0.001	0.22	<0.001	0.68
	***Tailgating***	-	-	0.12	<0.001	0.12	<0.001	1
	***MultiVeh***	0.47	<0.001	-	-	0.47	<0.001	-
	***SingleVeh***	0.22	<0.001	-	-	0.22	<0.001	-
	***MeanSpeed***	-	-	0.10	<0.001	0.10	<0.001	1
	***TrafficFlow***	0.27	<0.001	-0.06	0.044	0.21	<0.001	0.29
	***WetPav***	-	-	-0.06	0.009	-0.06	0.009	1
	***Distraction***	-	-	0.42	<0.001	0.42	<0.001	1
***FI***	***Speeding***	0.09	<0.001	0.09	<0.001	0.18	<0.001	0.50
	***Tailgating***	-	-	0.07	<0.001	0.07	<0.001	1
	***MultiVeh***	0.26	<0.001	-	-	0.26	<0.001	-
	***SingleVeh***	0.29	<0.001	-	-	0.29	<0.001	-
	***VRU***	0.42	<0.001	-	-	0.42	<0.001	-

Note: FI (Fatal and injury accidents), PDO (Property damage only accidents), VRU (Proportion of vulnerable road user involved accidents), SingleVeh (Proportion of Single-vehicle accidents), MultiVeh (Proportion of multi-vehicle accidents), WetPav (Wet pavement) VAF (Variance Accounted For).

### 5.2. Results of crash frequency by severity prediction

Reviewing Table 5, multi-vehicle accidents account for a high proportion of variation in PDO crash frequency. The mean speed was negatively associated with PDO crashes. The path model sheds light on the crash mechanism, partially explaining this association. Indeed, mean speed declines the prevalence of tailgating violations on the road, resulting in a decline in the risk of multi-vehicle accidents as the main predictor of PDO crash frequency. Indeed, non-severe accidents commonly occur in congested traffic due to elevated vehicle interactions, where the relative distance and freedom of maneuver are restricted. In line with this statement, the total effects imply that traffic flow is the main predictor of PDO crash frequency, while it is not the case in severe accidents (see Table 5). Accordingly, it is not surprising that the results indicated a negative association between mean speed and PDO crash frequency. Moreover, most recent findings, especially the real-time crash prediction models, confirm the current results [37, 38, 45]. These findings criticize previous studies which did not account for different effects that mean speed might have on crash risk for various severity levels. Hence, as the proportion of severe accidents might differ in databases, the magnitude and direction of the association would change in different studies accordingly [28]. While some previous studies evaluated the relationship between mean speed and crash risk, relying on certain crash severity levels due to the limited sample size [7, 11, 26]. On the other hand, the prevalence of speeding on the road was positively associated with the crash frequency of both severity levels, in line with previous findings [4].

As expected, VRU and single-vehicle accidents were the main crash types associated with the frequency of severe accidents. Moreover, distracted driving as the main predictor of these crash types was also the main explanatory variable of accidents with severe outcomes. Surprisingly, wet pavement was negatively associated with the frequency of severe accidents despite its positive relation with single-vehicle accidents. Meanwhile, this negative effect can be attributable to the inverse association of wet pavement conditions and VRU accidents as the main predictor of severe accidents. Nevertheless, the suppressor effect of wet pavement on crash severity has rarely been reported in the literature [93, 94], and the majority of studies have found a positive effect [95].

Overall, the results indicated that traffic flow, speeding, distracted driving, mean speed, and tailgating are the influential variables on PDO crash frequency, respectively. While distracted driving, traffic flow, speeding, mean speed, and wet pavement are the predictors of FI crash frequency. It is worth noting that human factors play a significant mediating role in the relationship between traffic and environmental characteristics with the frequency of PDO/FI accidents (0.16<VAF<1).

### 5.3. Detecting the unobserved heterogeneity

The FIMIX segmentation method was employed in an iterative framework to detect the potential unobserved heterogeneity between observations. The model selection criteria for selecting the number of homogenous sub-groups of data are detailed in Table 6. The information criteria indicated that the observations stem from a homogenous population since the one-segment scenario had the best fit. This denotes that the underlying crash mechanisms found in this research are stable over the two years in the study region. As the population of drivers during the peak periods of recreational travel is relatively unfamiliar with the roadway, the risk compensation effect and, accordingly, the magnitude of the path coefficients might differ in some subpopulations of the time series data. Nonetheless, scholars stated that the presence of unobserved heterogeneity in the crash time series data is less frequent compared with cross-sectional data. This can be attributed to the fact that crash records are aggregated for the same road entity over time. Although the unobserved temporal heterogeneity has been a major concern in the long-term crash time series, it is not almost the case for short periods [96].

**Table 6:** Model selection results.

		FIMIX-PLS path models			
***Number of segments (K)***		1	2	3	4
***The minimum size of segments***		731	284	91	88
***Criteria***					
	***Bayesian information criterion (BIC)***	16515	16536	16583	16871
	***Modified AIC 3 (AIC3)***	15839	16098	16026	16594
	***Consistent AIC (CAIC)***	16631	16730	16738	16948
	***Entropy statistic (EN)***	-	0.83	0.76	0.90

The EN statistic also indicates a potential overfitting issue for the four-segment scenario, as its value reaches the cut-off point of 0.9. The larger number of segments was not evaluated as the minimum acceptable sample size was not met (*N*_min_ = 44, for *K* = 5). The ten times rule concerning the minimum sample size implies that a minimum sample size of 50 is desired since the maximum number of paths entering an endogenous variable was five in the PLS-PM [77].

### 5.4. Practical applications

FIMIX-PLS segmentation, proposed in this study, suggests a new research direction in crash causal analysis. The previous methods failed to account for unobserved heterogeneity based on the structural model to unravel the heterogeneity between observations, which, in turn, results in different crash patterns within each data segment. Nonetheless, FIMIX-PLS not only detects these latent segments but also simultaneously estimates group-specific path coefficients. This, in turn, allows researchers to explore different crash mechanisms in each subpopulation of data without a-priori information on those latent classes. Meanwhile, it is not the only research gap that would be addressed by the latent class SEM approach.

The latent class analysis also has subsequent steps, termed Ex post analysis, that can identify the source of heterogeneity, proposing valuable insights to researchers [24]. After the FIMIX-PLS method illustrated the subgroups of data, the classification algorithms would be used to reproduce FIMIX-PLS partitions based on explanatory variables, like drivers’ behavioral differences, weather condition discrepancies, and road quality measures [97].

The results highlighted the contribution of VRU accidents to the frequency of severe outcome accidents since, in such cases, there is little or no external protective device that could absorb the impact of a road crash [98]. Meanwhile, the results are somehow exceptional for rural multilane highways, which are expected to be under high access control. This might be attributed to poor traffic facilities for the safe passage of pedestrians across the road, which deserves special attention from road safety policymakers.

The results also highlighted the crucial mediating role of drivers’ behavior in the causation of accidents. Multi-vehicle accidents accounted for a high proportion of total accidents, while tailgating and speeding were the most important predictors of such accidents. Hence, TV campaigns and driving-training programs should focus on highlighting “The two-second rule”, as has shown promising results elsewhere [99]. Moreover, automated enforcement cameras capable of detecting tailgating violations on Tehran’s highways are strongly recommended. Unfortunately, Iran police have not yet employed such cameras.

Distracted driving was the main predictor of VRU and single-vehicle accidents, which, in turn, were associated with the frequency of severe accidents. Hence, national distracted driving awareness campaigns would be influential in decreasing severe accidents [100]. Also, in-vehicle distraction and speeding detection devices in an incentive-based insurance program might influence declining those risky behaviors on the road [101]. Moreover, distracted driving was more frequent in less demanding conditions, namely, low traffic flow and high mean speed. Further research is needed to unravel the psychological processes explaining the study results.

### 5.5. Limitations

There are some limitations in this research, which should be considered when interpreting the results. First, the crash records in Iran are not in high resolution; hence it was impossible to link each crash record to its corresponding traffic-environmental features. Meanwhile, the aggregate daily records resulted in valuable and plausible insights. Second, many other potential crash contributing factors such as psychological factors (e.g., risk perception, overconfidence, traits, etc.), human factors (age, gender, driving skills, work schedule, etc.), roadway features (the presence of traffic enforcement cameras/shoulders/medians, lane widths, etc.), and the built environment (number of access points, land use, etc.) effects and their interactions were not investigated in the current study. Although it was tried to limit the study to relatively similar road entities, the potential biases that confounding variables such as roadway-related features might have in the speed-safety association should be considered. Moreover, as the risk compensation effect revealed a significant role in explaining the results, future studies would concentrate on the association of traffic-environmental features with operating speed and accidents, considering the psychological factors as potential mediators. Also, future work should include a richer dataset to ensure that these factors are well studied and reduce the potential for omitted variable bias. Employing the proposed causal analysis method on high-resolution datasets like that of the SHRP 2 Naturalistic Driving Study would bring further insights to the controversial topic of speed-safety association. Third, this study was conducted on a homogenous set of facilities with relatively similar design features. Accordingly, FIMIX-PLS only accounted for potential temporal unobserved heterogeneity, as the data stem from the same road entity over a two-year period. Future research would bring further insights to other potential heterogeneities using a diverse cross-sectional dataset containing different road classes.

## 6. Conclusion

The relationship between mean speed and crash likelihood has remained one of the most debated topics in the traffic safety field. The current inconclusive results can be attributed to the complex confounding, moderating, and mediating effects of explanatory variables on this association. This paper employed the PLS-PM method as a second-generation statistical method to account for such modeling complexities. Moreover, this study introduced FIMIX-PLS segmentation as a valuable instrument for detecting unobserved heterogeneity and evaluating the stability of the causal associations found between the variables of interest.

The following findings were made in this research: (1) The mean speed effects on the crash risk are entirely different for each crash type; hence, the distinct distribution of crash types in the different datasets would lead to inconsistent results. Accordingly, scholars should account for those unobserved effects when modeling the speed-crash risk association. Otherwise, such different effects might cancel each other and result in misleading conclusions. (2) The mean speed is positively and negatively associated with severe and PDO crash frequency, respectively. Conversely, speeding is positively correlated with the frequency of both crash severity levels. Hence, violating the speed limit is an important crash risk factor, whether the crash outcome is severe or not. On the other hand, the mean speed is a proxy variable that stands for the overall traffic flow state (e.g., the level of traffic congestion or proximity of adjacent vehicles). Accordingly, driving at speeds deemed unsafe for the corresponding condition leads to crashes, not necessarily the mean operating speed. (3) The prevalence of speed violations is positively associated with highway mean speed which can be attributed to the speed adaptation effect. Hence, unraveling the psychological process that drives individuals to adapt the mean operating speed or speeds of adjacent vehicles would help to reduce speeding on the road. Moreover, highway mean speed is lower during higher traffic flows and adverse weather conditions, which can be related to the risk compensation effect. Hence, increasing the risk perception skills of drivers through risk awareness campaigns would be help decline the risk-taking behavior of drivers in conditions with lower workloads. (4) Distracted driving and wet pavement condition are the main predictors of single-vehicle and VRU accidents, while speeding and tailgating are the most influential factors in multi-vehicle accidents. (5) The lower the mean speed and the higher the traffic flow, the higher the percentages of tailgaters on the road; thus, the higher the likelihood of multi-vehicle accidents, which, in turn, are associated with a higher frequency of PDO crashes. (6) The higher the mean speed and the lower the traffic flow, the higher the prevalence of distracted driving on the road; thus, the higher the likelihood of single-vehicle and VRU accidents, which, in turn, are associated with a higher frequency of severe accidents.

Conclusively, two distinct crash mechanisms are evident in the study region, which are related to severe and non-severe crash outcomes. The first mechanism implies that in the high-density traffic state (e.g., the lower mean speeds and the higher traffic flows), the frustrated drivers tailgate the leading vehicle and violate the safe spacing, which in turn results in a higher frequency of non-severe crashes. Hence, to eliminate the total number of crashes which are almost of the non-severe type, policymakers should install tailgating detection enforcement cameras on Tehran highways. Targeting this crucial mediator in the speed-safety association would decline the PDO crash frequency. On the other hand, the other crash mechanism implies that distracted driving is a critical mediator of the association between higher speeds and severe outcome crashes. Indeed, in the low-density traffic state, which is associated with a lower driving workload, the higher prevalence of distracted driving on the road results in a higher frequency of run-of-the-road, roll over, and VRU crashes. Hence, investigating the psychological processes like the risk compensation mechanism, which might explain the results, would bring new insight into potential cognitive interventions that might reduce distracted driving on empty roads.

The second-generation statistical modeling approach, with its ability to account for complex indirect associations between crash risk factors, not only increases our knowledge of the distinct crash mechanisms but also suggests efficient interventions by identifying the key policy-sensitive mediators. Moreover, the FIMIX-PLS segmentation method would help researchers to uncover the latent distinct crash mechanisms in the sub-groups of crash datasets, leading to a more comprehensive perspective in traffic safety studies.

## Supporting information

S1 File The crash dataset used in this study. (XLSX)

S2 File Poisson and normal distributions goodness-of-fit tests. (PDF)

S1 Appendix Partial Least Square Structural Equation Modeling (PLS-SEM). (PDF)

S2 Appendix Finite Mixture Partial Least Square (FIMIX-PLS) segmentation. (PDF)
