# Supplementary material for: Investigating speed-safety association: Considering the unobserved heterogeneity and human factors mediation effects
Source: PLoS One. 2023 Feb 21;18(2):e0281951. doi: 10.1371/journal.pone.0281951 (PMC9943019; doi:10.1371/journal.pone.0281951)
Supplement: S2 File — (PDF) [file pone.0281951.s002.pdf]

Investigating speed-safety association: Considering the unobserved heterogeneity, and human factors mediation effects

Supplemental material

S2 File. Poisson and normal distributions goodness-of-fit tests.

The Chi-square test is used to test the null hypothesis that the set of data comes from a Poisson distribution. (H0: the data is Poisson distributed)

Poisson Goodness-of-Fit Test: FI (Fatality/injury crashes)

Descriptive Statistics

N	Mean
730	8.97671

Observed and Expected Counts for FI

FI	Poisson Probability	Observed Count	Expected Count	Contribution to Chi-Square
<=2	0.006350	64	4.6352	760.300
3	0.015229	30	11.1170	32.074
4	0.034176	52	24.9485	29.332
5	0.061358	53	44.7911	1.504
6	0.091798	48	67.0128	5.394
7	0.117721	68	85.9363	3.744
8	0.132093	56	96.4282	16.950
9	0.131752	53	96.1787	19.385
10	0.118270	58	86.3369	9.301
11	0.096516	42	70.4565	11.493
12	0.072199	44	52.7056	1.438
13	0.049855	25	36.3941	3.567
14	0.031967	35	23.3357	5.830
15	0.019130	23	13.9652	5.845
16	0.010733	15	7.8351	6.552
17	0.005667	11	4.1373	11.384
>=18	0.005186	53	3.7859	639.743

3 (17.65%) of the expected counts are less than 5.

Chi-Square Test

Null hypothesis H_0: Data follow a Poisson distribution
Alternative hypothesis H_1: Data do not follow a Poisson distribution

DF	Chi-Square	P-Value
15	1563.84	0.000

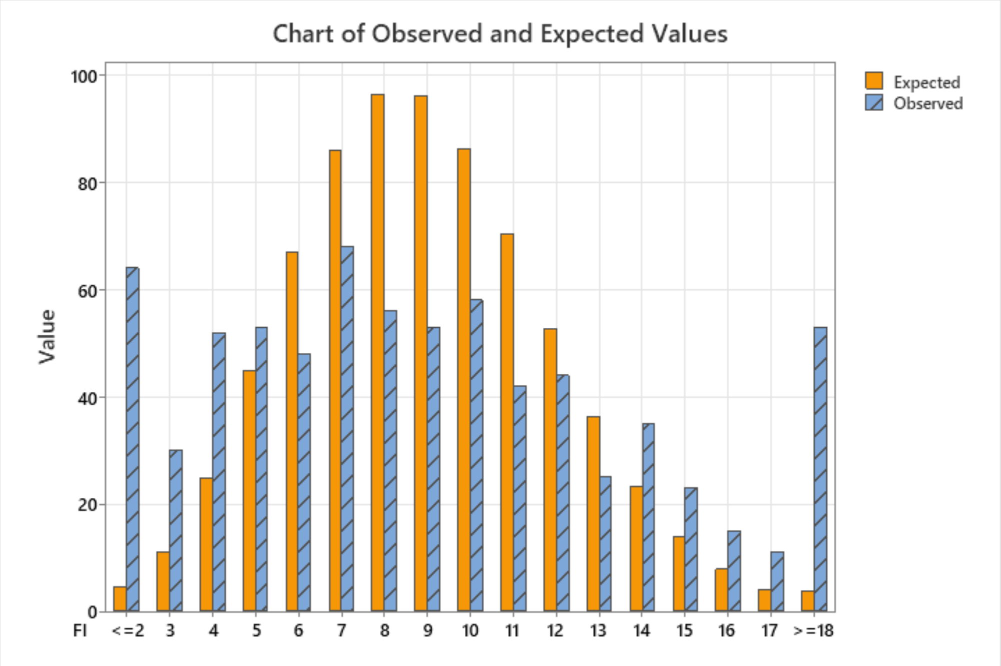
Chart of Observed and Expected Values
Expected
Observed
Value
FI
<=2
3
4
5
6
7
8
9
10
11
12
13
14
15
16
17
>=18

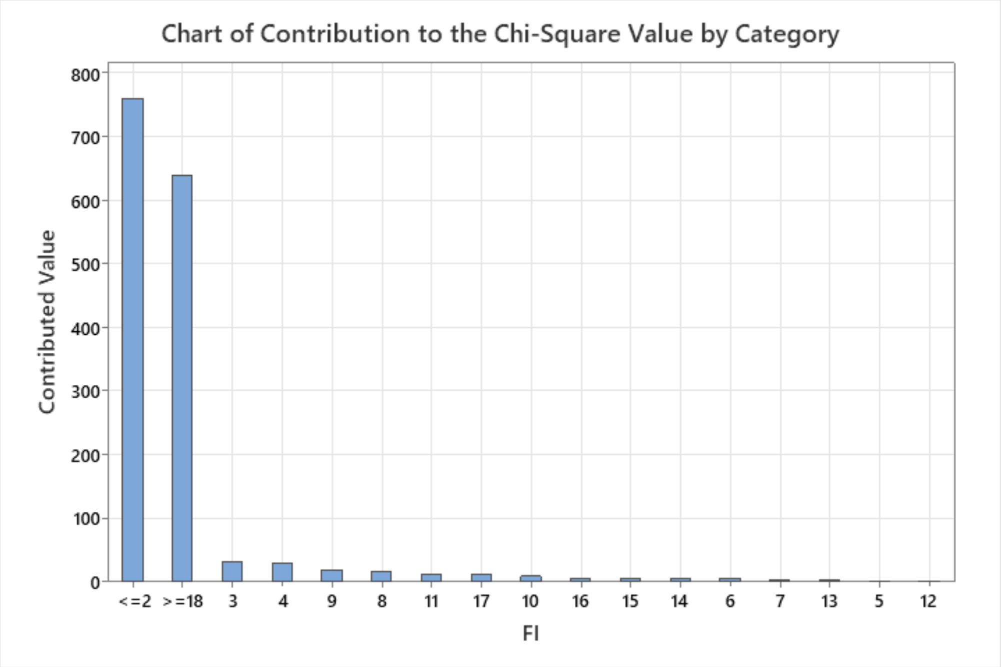
Chart of Contribution to the Chi-Square Value by Category
Contributed Value
<=2
>=18
3
4
9
8
11
17
10
16
15
14
6
7
13
5
12
FI

Poisson Goodness-of-Fit Test: PDO (property damage only crashes)

Descriptive Statistics

N	Mean
730	23.4589

Observed and Expected Counts for PDO

PDO	Poisson Probability	Observed Count	Expected Count	Contribution to Chi-Square
<=11	0.003428	66	2.502	1611.18
12 - 13	0.010547	20	7.700	19.65
14 - 15	0.029156	41	21.284	18.26
16 - 17	0.062058	46	45.302	0.01
18 - 19	0.104795	52	76.500	7.85
20 - 21	0.143779	66	104.959	14.46
22 - 23	0.163407	69	119.287	21.20
24 - 25	0.156329	81	114.120	9.61
26 - 27	0.127610	57	93.155	14.03
28 - 29	0.089913	56	65.637	1.41
30 - 31	0.055234	56	40.321	6.10
32 - 33	0.029842	40	21.784	15.23
34 - 35	0.014289	17	10.431	4.14
36 - 37	0.006106	19	4.457	47.45
>=38	0.003506	44	2.559	670.96

3 (20.00%) of the expected counts are less than 5.

Chi-Square Test

Null hypothesis	H_0: Data follow a Poisson distribution
Alternative hypothesis	H_1: Data do not follow a Poisson distribution

DF	Chi-Square	P-Value
13	2461.54	0.000

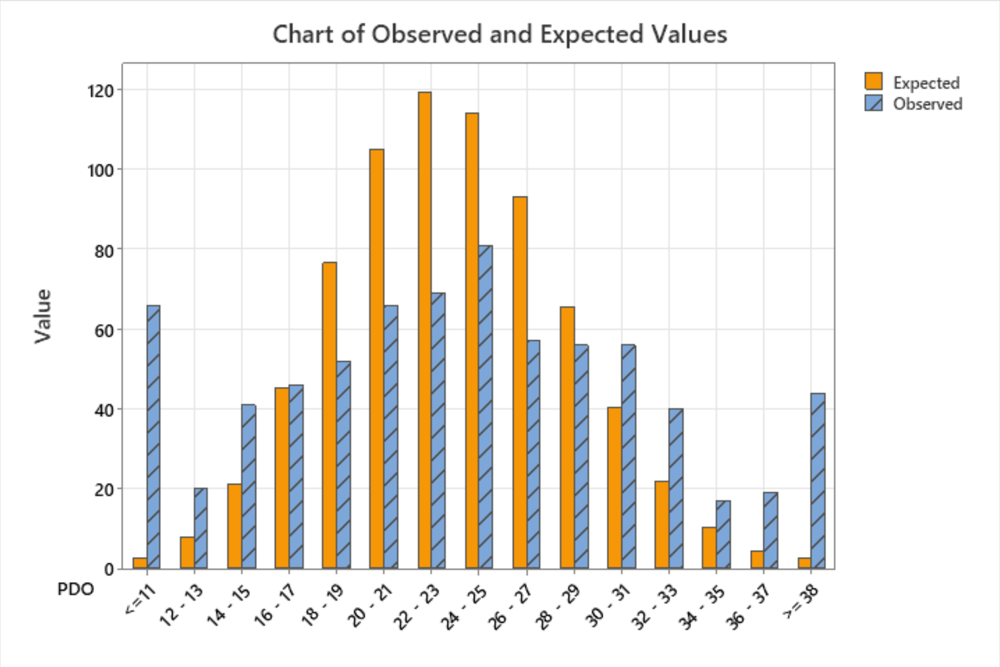

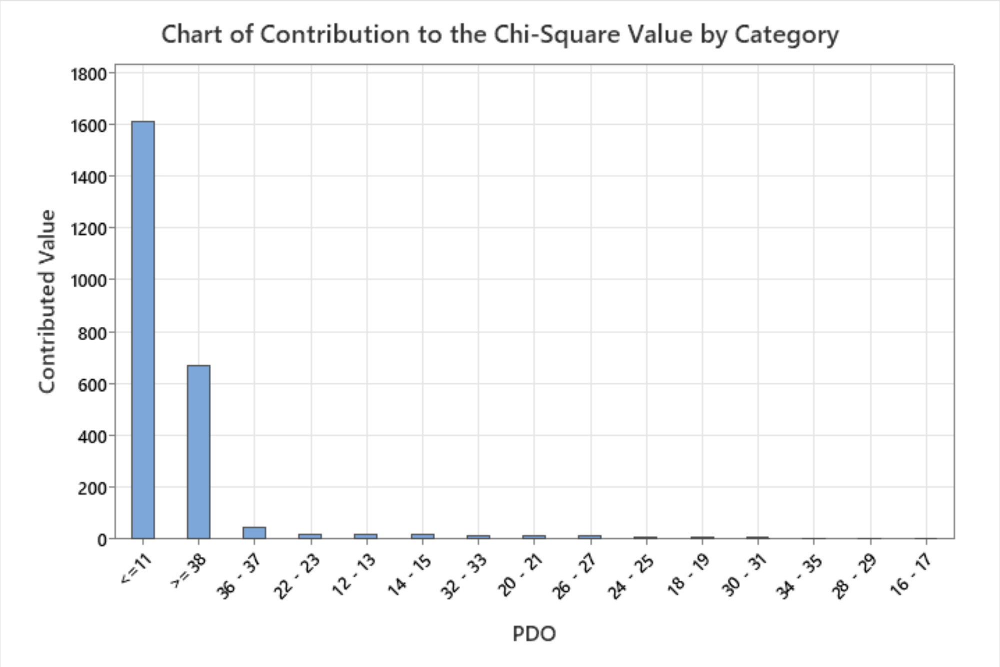

- The data does not fit to Poisson distribution. This is more evident for the less severe outcomes (PDO) which are not so rare.

The Kolmogorov-Smirnov test is used to test the null hypothesis that the set of data comes from a normal distribution. (H0: the data is normally distributed)

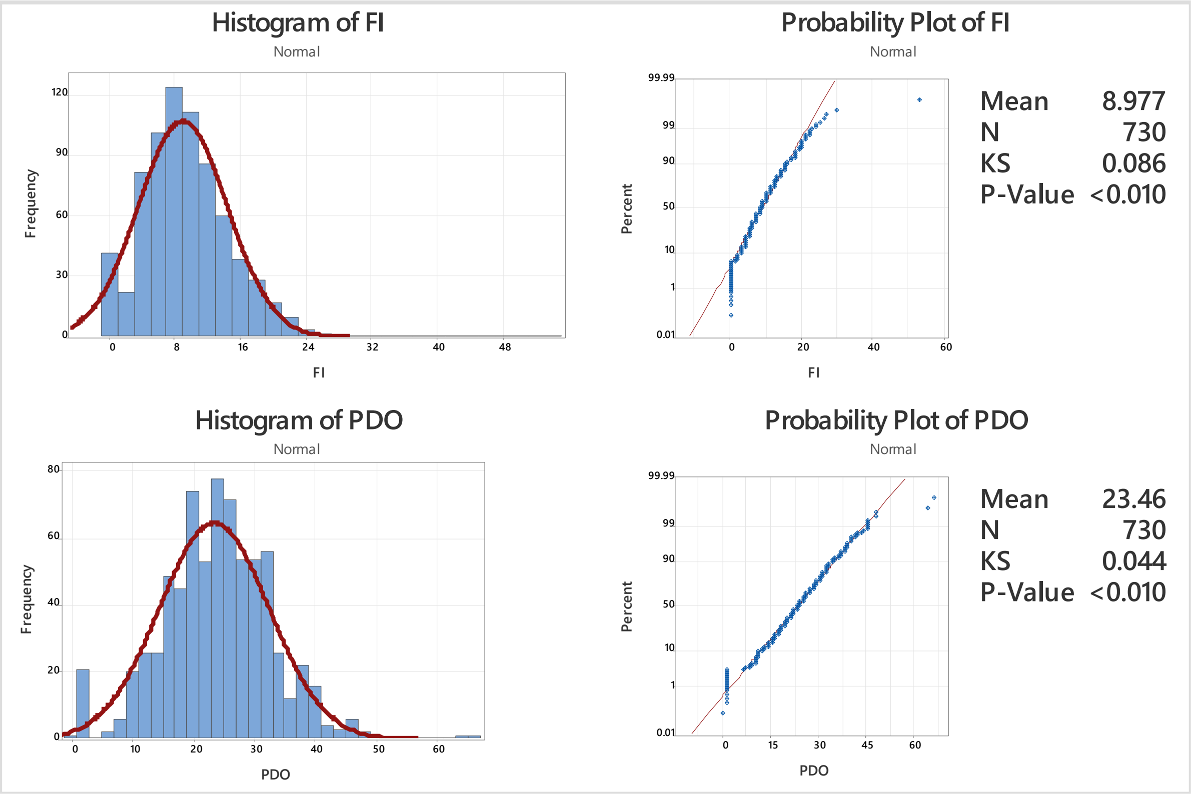

- The data does not fit exactly to normal distribution but it is slightly skewed to the right. This is more evident for the severe outcomes (FI) which are rare.
