# Supplementary material for: Investigating speed-safety association: Considering the unobserved heterogeneity and human factors mediation effects
Source: PLoS One. 2023 Feb 21;18(2):e0281951. doi: 10.1371/journal.pone.0281951 (PMC9943019; doi:10.1371/journal.pone.0281951)
Supplement: S1 Appendix — (PDF) [file pone.0281951.s003.pdf]

Investigating speed-safety association: Considering the unobserved heterogeneity, and human factors mediation effects

Supplemental material

S1 APPENDIX. Partial Least Square Structural Equation Modeling (PLS-SEM)

The SEM approach consists of two submodels: a structural (path/inner) model and a measurement (outer) model [1]. In the measurement model analysis, PLS method considers the associations between observed variables (e.g., indicators) with their corresponding latent construct. Indeed, the measurement model evaluates how well a linear combination of the indicators can estimate their corresponding latent construct [2]. Then, PLS examines the relationships in the structural model between manifest or latent variables based on an iterative algorithm, which relies on ordinary least square (OLS) estimation so that the R^2 values of endogenous variables are maximized [2]. The estimated path coefficients, depicted on the vectors in the inner model indicate the strength of the hypothesized associations between variables. Eq. (1) and Eq. (2) illustrate the model formulation for the measurement and structural models, respectively [3].

$$v_i = \lambda_i F_i + e_i \tag{1}$$

Where: v_i is a vector of indicators; F_i is a vector of latent variables; λ_i and $e_i$are the vectors of parameters and measurement errors, respectively.

$$F_i^{**} = \beta_i F_i^{*} + \Gamma_i F_i + d_i \tag{2}$$

Where: the endogenous variable F_i^{**} is a function of the effects of exogenous variable F_i and the endogenous effects of mediating variable F_i^{*} plus disturbance terms d_i. Γ_i and β_i are parameter vectors.

Bibliography

1. Vinzi VE, Trinchera L, Amato S. PLS path modeling: from foundations to recent developments and open issues for model assessment and improvement. Handbook of partial least squares: Springer; 2010. p. 47-82.
2. Hair Jr JF, Hult GTM, Ringle CM, Sarstedt M. A primer on partial least squares structural equation modeling (PLS-SEM): Sage publications; 2021.
3. Kim K, Pant P, Yamashita E. Measuring influence of accessibility on accident severity with structural equation modeling. Transportation research record. 2011;2236(1):1-10.
