# Supplementary material for: Investigating speed-safety association: Considering the unobserved heterogeneity and human factors mediation effects
Source: PLoS One. 2023 Feb 21;18(2):e0281951. doi: 10.1371/journal.pone.0281951 (PMC9943019; doi:10.1371/journal.pone.0281951)
Supplement: S2 Appendix — (PDF) [file pone.0281951.s004.pdf]

Investigating speed-safety association: Considering the unobserved heterogeneity, and human factors mediation effects

Supplemental material

S2 APPENDIX. Finite Mixture Partial Least Square (FIMIX-PLS) segmentation

FIMIX-PLS follows a latent class approach by assuming that data stem from several subpopulations. Each data segment is modeled distinctively, and the overall observations are a mixture of the segments [1]. That is, each observation is regarded as a realization of the mixture density with K segments [2], where:

$$f_{i|k}(x_i|\theta_k) = \sum_{k=1}^{K} \rho_k \, f_{i|k}(x_i) \qquad (1)$$

With $f_{i|k}(.)$ being a density function, $\rho_k$the relative segment size, and θ_k the kth segment's vector of unknown parameters.

Drawing on this concept, FIMIX-PLS has the following function form:

$$\eta_i = \sum_{k=1}^{K} \rho_k \, f_{i|k}(\eta_i|\xi_i, B_k, \Gamma_k, \Psi_k) \qquad (2)$$

Where $\Psi_k$is the matrix of the kth segment's regression variances in the structural model, $\Gamma_k(\mathrm{B}_k)$ is the path coefficient matrix of the exogenous (endogenous) constructs, and $\eta_i$is the matrix of endogenous latent variable scores. Substituting the density function leads to:

$$\eta_i = \sum_{k=1}^{K} \rho_k \left[\frac{1}{(2\pi)^{Q/2}\sqrt{|\Psi_k|}}\right] exp\{-0.5((I - B_k)\eta_k + (-\Gamma_k)\xi_i)\hat{A}'{\Psi_k}^{-1}((I - B_k)\eta_k + (-\Gamma_k)\xi_i)\} \qquad (3)$$

Where I is the identity matrix and Q denotes the number of endogenous constructs in the structural model.

FIMIX-PLS estimates the parameters using expectation maximization (EM) algorithm [3], which maximizes the log-likelihood function of (3), as follows:

$$LnL = \sum_{i=1}^{N}\sum_{k=1}^{K} z_{ik}\, ln(f(\eta_i|\xi_i, B_k, \Gamma_k, \Psi_k)) + \sum_{i=1}^{N}\sum_{k=1}^{K} z_{ik}\, ln(\rho_k) \qquad (4)$$

Where $z_{ik}$is 1 if observation i belongs to segment k, 0 else. $\rho_k$is the relative segment size and the ξ_i, B_k, Γ_k, and $\Psi_k$are as defined in Eq. (2). The expected values $E(z_{ik}) = \mathrm{P}_{ik}$ are calculated based

on Bayes' theorem [4]. The algorithm stops after two consecutive iterations make no significant improvement in the LnL (Eq. (4)).

The calculated segment membership probabilities P_{ik} is then employed to compute the relative segment sizes:

$$\rho_k = \sum_{i=1}^{N} \frac{P_{ik}}{N} \qquad (5)$$

Similarly, segment-specific parameter estimates, namely, indicator loadings (weights) and path coefficients, are calculated utilizing weighted regressions using P_{ik} as observation weights.

Bibliography

1. McLachlan GJ, Lee SX, Rathnayake SI. Finite mixture models. Annual review of statistics and its application. 2019;6:355-78.
2. Sarstedt M, Radomir L, Moisescu OI, Ringle CM. Latent class analysis in PLS-SEM: A review and recommendations for future applications. Journal of Business Research. 2022;138:398-407.
3. Dempster AP, Laird NM, Rubin DB. Maximum likelihood from incomplete data via the EM algorithm. Journal of the Royal Statistical Society: Series B (Methodological). 1977;39(1):1-22.
4. Rigdon EE, Ringle CM, Sarstedt M. Structural modeling of heterogeneous data with partial least squares. Review of marketing research. 2010.
